# Postoperative Structural Brain Changes and Cognitive Dysfunction in Patients with Breast Cancer

**DOI:** 10.1371/journal.pone.0140655

**Published:** 2015-11-04

**Authors:** Chiho Sato, Atsushi Sekiguchi, Masaaki Kawai, Yuka Kotozaki, Rui Nouchi, Hiroshi Tada, Hikaru Takeuchi, Takanori Ishida, Yasuyuki Taki, Ryuta Kawashima, Noriaki Ohuchi

**Affiliations:** 1 Department of Functional Brain Imaging, Institute of Development, Aging and Cancer (IDAC), Tohoku University, Sendai, Japan; 2 Division of Surgical Oncology, Graduate School of Medicine, Tohoku University, Sendai, Japan; 3 Division of Medical Neuroimage Analysis, Department of Community Medical Supports, Tohoku Medical Megabank Organization, Tohoku University, Sendai, Japan; 4 Department of Adult Mental Health, National Institute of Mental Health, National Center of Neurology and Psychiatry, Tokyo, Japan; 5 Department of Breast Oncology, Miyagi Cancer Center Hospital, Miyagi, Japan; 6 Division of Clinical Research, Medical-Industry Translational Research Center, Fukushima Medical University School of Medicine, Fukushima, Japan; 7 Creative Interdisciplinary Research Division, Frontier Research Institute for Interdisciplinary Science, Tohoku University, Sendai, Japan; 8 Department of Advanced Brain Science, Smart Ageing International Research Center, IDAC, Tohoku University, Sendai, Japan; 9 Human and Social Response Research Division, International Research Institute of Disaster Science, Tohoku University, Sendai, Japan; 10 Division of Developmental Cognitive Neuroscience, IDAC, Tohoku University, Sendai, Japan; 11 Department of Nuclear Medicine and Radiology, Institute of Development, Aging and Cancer, Tohoku University, Sendai, Japan; Banner Alzheimer's Institute, UNITED STATES

## Abstract

**Objective:**

The primary purpose of this study was to clarify the influence of the early response to surgery on brain structure and cognitive function in patients with breast cancer. It was hypothesized that the structure of the thalamus would change during the early response after surgery due to the effects of anesthesia and would represent one aspect of an intermediate phenotype of postoperative cognitive dysfunction (POCD).

**Methods:**

We examined 32 postmenopausal females with breast cancer and 20 age-matched controls. We assessed their cognitive function (attention, memory, and executive function), and performed brain structural MRI 1.5 ± 0.5 days before and 5.6 ± 1.2 days after surgery.

**Results:**

We found a significant interaction between regional grey matter volume (rGMV) in the thalamus (*P* < 0.05, familywise error (FWE), small volume correction (SVC)) and one attention domain subtest (*P* = 0.001, Bonferroni correction) after surgery in the patient group compared with the control group. Furthermore, the changes in attention were significantly associated with sevoflurane anesthetic dose (*r* ^2^ = 0.247, β = ‒0.471, *P* = 0.032) and marginally associated with rGMV changes in the thalamus (*P* = 0.07, FWE, SVC) in the Pt group.

**Conclusion:**

Our findings suggest that alterations in brain structure, particularly in the thalamus, may occur shortly after surgery and may be associated with attentional dysfunction. This early postoperative response to anesthesia may represent an intermediate phenotype of POCD. It was assumed that patients experiencing other risk factors of POCD, such as the severity of surgery, the occurrence of complications, and pre-existing cognitive impairments, would develop clinical POCD with broad and multiple types of cognitive dysfunction.

## Introduction

As a result of the long-term survival of patients with breast cancer due to improved screening and treatment, the quality of life (QOL) of such patients has received increased attention. Maintaining QOL is now critical, particularly for patients in industrialized countries, because the number of patients with breast cancer worldwide is increasing by >1,000,000 annually [1,2]. Cognitive dysfunction as a result of cancer therapy affects the QOL of breast cancer patients [3,4]. Although the influence of chemotherapy on cognitive dysfunction has been well characterized [5], patients often experience cognitive dysfunction even before starting chemotherapy [6,7]. In the current study, we focused on cognitive dysfunction in patients with breast cancer after surgery, which is known as postoperative cognitive dysfunction (POCD), and assessed its potential impact on QOL [8,9].

The clinical features of POCD, such as the rates of complication and risk factors, have been elucidated, but its neuropathology remains unclear due to the gap between clinical and pre-clinical observations [10]. The clinical features of POCD encompass a variety of cognitive impairments [11], and several risk factors including advanced age, long duration of anesthesia, the level of surgical severity, the occurrence of complications, and pre-existing cognitive impairments [12–14]. POCD has been defined by the International Study of Post-Operative Cognitive Dysfunction (ISPOCD) as the deterioration of two or more cognitive functions from multiple cognitive domains [12,15]. Thus, in clinical terms, POCD comprises dysfunction in a variety of cognitive domains, and it seems to be associated with multiple risk factors [10]. In contrast, pre-clinical studies have demonstrated that the fundamental neuropathology underlying postoperative cognitive decline can be caused by a single risk factor, such as anesthesia or inflammation [16–18]. Thus, well-controlled clinical studies are required to better characterize the roles that individual risk factors play in clinical situations.

To address this issue, the present study used neuroimaging methods to assess the early responses of patients to general anesthesia following a low invasive surgery. To assess the responses to general anesthesia as a single risk factor for POCD, other risk factors were controlled using three criteria: 1) patients underwent surgery for breast cancer, which was selected because this procedure is unlikely to induce systemic inflammation; 2) clinical assessments for the study occurred prior to the initiation of adjuvant therapy; and 3) no patients had pre-existing cognitive impairments and no complications after surgery. Also, neuroimaging is a powerful tool for assessing common brain structural changes which could be identified as an intermediate phenotype, even if the cognitive changes are not apparent enough to be classed as a syndrome [19]. However, few POCD neuroimaging studies have been reported. Chen et al. reported that a smaller regional grey matter volume (rGMV) in the hippocampus before surgery was a risk factor for POCD at postoperative day 4 [20]. Kline et al. performed a longitudinal study demonstrating reductions in the total gray matter and regional hippocampus volumes 5–9 months after surgery [21]. Because of the high incidence of POCD soon after surgery [13], assessment of cognitive decline and structural brain changes in the early postoperative period may allow the detection of one neuropathological aspect of POCD.

The aim of the present study was to clarify the influence of the early response to surgical operations on brain structure and cognitive function in patients with breast cancer. We recruited 32 postmenopausal females with early-stage breast cancer (Pt), and 20 age-matched postmenopausal healthy controls (HC). We assessed cognitive functions (attention, memory, and executive functions), and collected brain structural magnetic resonance (MR) images 1.5 ± 0.5 days before (pre) and 5.6 ± 1.2 days after (post) surgery in both the Pt and HC groups.

Pre-clinical studies suggest that general anesthesia plays a key role in the development of POCD symptoms [10]. Although general anesthesia acts on various regions in brain, the thalamus seems to be a major target. For example, the volatile and intravenous anesthetics sevoflurane [22], propofol [23], and fentanyl [24] reduced cerebral blood flow, and sevoflurane [25] reduced regional glucose metabolism in the thalamus. The thalamus plays an important role in coordinating the flow of information in the brain, and integrating broad cognitive processes [26,27], including incoming sensory impulses of pain [28], the regulation of arousal and sleep [29,30], and anesthetic-induced loss of consciousness [31]. Therefore, we hypothesized that a reduction in thalamic rGMV shortly after surgery and cognitive dysfunction associated with a reduction in thalamic rGMV would be observed.

## Materials and Methods

### Participants

**Fig 1:**
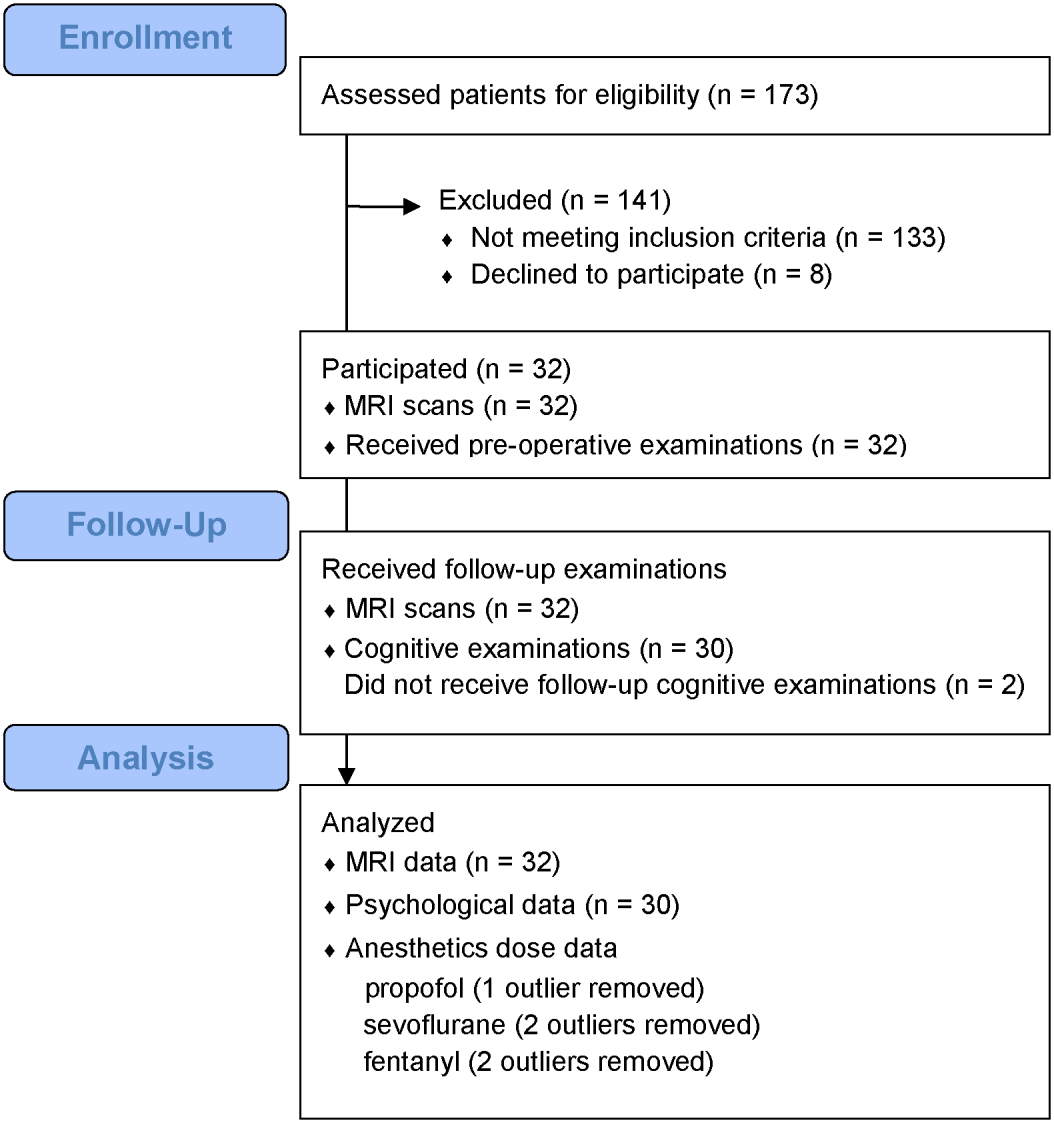
Enrollment, Participation, Follow-up, and Analysis of the patient group.

Patients with breast cancer who were planned to receive surgical operation were recruited from Tohoku University hospital between February 2012 and April 2013. The inclusion criteria were as follows: 1) female gender to minimize gender-based brain differences, and 2) post-menopausal females aged ≤ 80 years to reduce the influence of hormonal status on cognition. The following exclusion criteria were applied: 1) any history of cancer therapy, including chemotherapy and hormonal therapy; 2) any history of neurological disorders, traumatic brain injury, or psychiatric disorders; 3) any history of substance abuse and dependence; 4) diabetes, uncontrolled hypertension, or any organ failure; and 5) any contraindication to undergoing an MRI scan. One hundred and seventy-three women who were newly diagnosed with breast cancer were screened for eligibility. One hundred and thirty-three patients did not meet the study’s eligibility criteria. The most common reasons for ineligibility were prememopausal status and receiving neoadjuvant therapy. Eight patients declined to participate because they were either uninterested, too busy, or lived too far away. Thirty two patients were recruited and completed MRI scans. Thirty patients completed the cognitive examinations and 2 patients did not undergo a post-operative examination, even in the absence of physical complications after surgery. Thirty two MRI data and 30 psychological data were analyzed in patients (Fig 1). All clinical information was collected from medical records. We also recruited 20 healthy subjects who resided in the same geographical areas as the patients using advertisements in a local free newspaper in January 2013. The inclusion and exclusion criteria were identical to those for cancer patients, except for the requirement of a history of breast cancer surgery. Nineteen of the 20 healthy controls completed the study protocol, and one control participant could not undergo MRI scanning due to a metal finger ring that could not be removed at time on the scan. All subjects reported their height, weight, education history, and lifetime alcohol intake using self-administered questionnaires at the time of entry to the study. The questionnaires for alcohol intake included the duration of alcohol intake, the frequency of alcohol consumption per week, and the amount and types of alcohol consumed per day. Written informed consent was obtained from all subjects. The Ethics Committee of the Tohoku University Graduate School of Medicine approved this study. This study was conducted between February 2012 and May 2013 in Sendai city, Miyagi prefecture, Japan.

### Protocol

Assessments were performed during hospitalization. Preoperative assessment was performed 1 or 2 days before surgery (pre), and postoperative assessment was performed within 1 week of surgery (post). The healthy group underwent two assessments, with a 1-week interval. Because the present study was observational, there were no restrictions on the types of anesthesia used. The study was registered in the University Hospital Medical Information Network (UMIN) Clinical Trial Registry (UMIN 000007287).

### Psychological measurements

To evaluate the influence of surgery, we assessed a broad range of cognitive functions in five categories (attention, processing speed, memory, executive function, and working memory).

Attention was assessed using a Digit Cancellation Task (D-CAT) [32]. The test sheet consisted of 12 rows of 50 digits, which each contained five sets of the numbers 0–9 arranged in a random order. Participants were instructed to search for target number(s) with a slash mark as quickly and as accurately as possible for 1 min. Three trials were used: first with a single target number (6), second with two target numbers (9 and 4), and third with three (8, 3, and 7).

Processing speed was assessed using digit symbol coding, which is a subtest within the Wechsler Adult Intelligence Scale, third edition (WAIS-III) [33]. This test used a key, which shows a series of symbols paired with numbers; participants were instructed to draw each symbol under its corresponding number for 2 min.

Memory was measured by Logical Memory I (immediate) and II (30-min delay) tests, which are subtests of the Japanese version of the Wechsler Memory Scale-Revised (WMS-R) test [34–36]. Participants memorized two short, paragraph-length stories (stories A and B). The stories were scored in terms of the number of story units recalled, as specified in the WMS-R scoring protocol.

Executive function and processing speed were measured using the Stroop test [37,38], including response inhibition and impulsivity; we used Stroop 2 (the reverse Stroop task) and Stroop 4 (the Stroop task). To measure processing speed, we used Stroop 1 and 3.

Working memory was measured using digital span—backwards, which is a subtest of WAIS-III [33]. Participants were instructed to memorize numbers, and repeat them in reverse order.

We also evaluated quality of life using the Japanese version of the World Health Organization Quality of Life Instrument—Short Version; WHOQOL-BREF (WHO/QOL-26) [39].

### Image acquisition

All MRI data were acquired using a 3-T Philips Intera Achieva scanner (Best, Netherlands). A magnetization-prepared rapid gradient echo (MPRAGE) sequence was used to collect high-resolution T1-weighted structural images (240 × 240 matrix; 6.5-ms repetition time; 3-ms echo time; 24-cm field of view; 162 slices; 1.0-mm slice thickness).

### Voxel-based morphometry analysis (VBM)

VBM was performed to investigate the structural changes due to surgery. The structural data were pre-processed using statistical parametric mapping software (SPM8; Wellcome Department of Cognitive Neurology, London, UK) implemented in Matlab (Mathworks Inc., Natick, MA, USA). The present study employed diffeomorphic anatomical registration through exponentiated lie (DARTEL) algebra, which is an improved VBM method for registration that can more accurately achieve inter-subject brain image registration [40]. This procedure is advantageous for longitudinal data because rigid inter-subject registration data across different time points can be explored in a linear way for local differences.

To begin with, T1-weighted structural images of each individual were segmented into six tissue sections using the new segmentation algorithm implemented in SPM8. Default parameters were used for this new segmentation process, except for affine regularization, which was performed using the International Consortium for Brain Mapping (ICBM) template for East Asian brains. We then proceeded to the DARTEL registration process implemented in SPM8. During this process, we used DARTEL-imported images of the two-tissue probability map (TPM) of gray and white matter created using the abovementioned segmentation process. First, the template for the DARTEL procedures was created using T1WI data from the pre-scan of all participants in the study. Next, we used this template to perform DARTEL (using default parameters) for the pre- and post-scan T1WI of all subjects. The resulting images were then normalized spatially to the Montreal Neurological Institute (MNI) space to obtain images with 1.5 × 1.5 × 1.5-mm^3^ voxels. In addition, we performed a volume change correction (modulation) by modulating each voxel with the Jacobian determinants derived from the spatial normalization, which enabled regional differences in the absolute amount of brain tissue to be determined [41]. Subsequently, all images were smoothed by convolution with an isotropic Gaussian kernel of 8-mm full-width at half-maximum (FWHM).

### Statistical analysis

Demographic differences for continuous variables (such as age) between the Pt and HC groups were calculated using two sample *t*-tests. To examine the general deterioration of cognitive function and QOL soon after surgery, we compared Pt with HC using analysis of covariance (ANCOVA) models. We used the post score as the dependent variable, the group (Pt, HC) as the categorical variable, and the pre score and age as covariates. Statistical significance was assessed at *P* < 0.0045 using Bonferroni corrections for comparison of multiple psychological measurements (*P* < 0.05/11). The results that differed significantly between groups were subsequently subjected to the correlation analyses described below.

To determine whether anesthesia was associated with the cognitive functions, which deteriorated in the Pt group compared to the HC group after surgery, we performed multiple regression analysis in the Pt group. Because the sample size of this analysis was relatively small, we used the change ratio of cognitive scores, which was obtained by dividing the post—pre scores by the pre scores, as dependent variables to keep the number of covariates appropriately and include pre-surgery cognitive performance in the analyses. We analyzed using the change ratio of differential cognitive measures as dependent variables, the doses of sevoflurane and fentanyl as independent variables, and age, height, and weight as covariates. Although we also used propofol for general anesthesia, we examined the correlation only with sevoflurane and fentanyl, because there were small individual, body-size-dependent differences in the propofol dose due to bolus usage. The anesthetics dose data that were 1.5 SD more or less than the mean were excluded from the analysis as outliers. The data from propofol, sevoflurane and fentanyl doses had one, two, and two patients as outliers, respectively, who were all different individuals. In addition, as described before, two patients did not undergo a post-operative examination. Therefore, we performed correlation analyses between psychological and anesthetics data, including sevoflurane and fentanyl doses from 28 patients, respectively. The threshold for statistical significance was set at *P* < 0.05 (one-tailed), with Bonferroni corrections for multiple comparisons for the amount of anesthetics (sevoflurane and fentanyl), with a strong priori hypothesis that the cognitive function would deteriorate due to the use of anesthetics. All statistical analyses were performed using SPSS 20 (SPSS, Chicago, IL).

Differences in rGMV were assessed as a group (Pt/HC) by time (pre/post) interaction using an analysis of covariance (ANCOVA) model on SPM8. The analyses were performed using age as a covariate. Small volume correction (SVC) was applied to one ROI based on the hypothesis (thalamus) using anatomical masks from the “Human aal atlas” within the Wake Forest University PickAtlas 3.04 (http://fmri.wfubmc.edu/software/PickAtlas) [42,43]. The ROI was defined from bilateral hemispheres. A significance level was set at *P* = 0.05 corrected for multiple comparisons (voxel-level family-wise error). In the analyses, we included only voxels that showed GMV values >0.10 to avoid possible partial volume effects around the borders between grey and white matter, as well as between grey matter and cerebrospinal fluid. Next, multiple regression analyses were performed for the patient group within the ROI (thalamus) to determine whether the reductions in rGMV were associated with the cognitive decline, and with anesthesia. To verify the relationship between the reduced rGMV and cognitive decline, the change ratios of the differential cognitive measures, which differed significantly between the Pt and HC groups, were included as dependent variables. The post-pre rGMV was used independent variables and age was treated nuisance covariates. To verify the relationship between the reduced rGMV and anesthesia, the post—pre rGMV was included as a dependent variable, the doses of the differential anesthetics were used as independent variables, and age were treated as nuisance covariates. Again, we excluded all voxels with GMV values >0.10. The significance level was set at *P* = 0.05 corrected for multiple comparisons (voxel-level family-wise error).

## Results

### Participant demographics and clinical data

No significant differences (*P* < 0.05) were found between the Pt and HC groups in terms of demographic characteristics (Table 1). The clinical data describing surgery are presented in Table 2. All surgeries were performed under general anesthesia. Although there were no restrictions on the type of anesthesia used in the current protocol, the same combination of anesthetics were used (propofol, sevoflurane, and fentanyl), except for in two individuals (remifentanil or nitrous oxide was added to the above combination of anesthetics). No patients experienced clinical complications, including delirium.

**Table 1:** Demographic characteristics of patients and controls subjects.

	Pt; *n* = 30	HC; *n* = 19	*P* value
Age (years)	60 ± 7	59 ± 5	0.69
Height (cm)	158 ± 4	157 ± 6	0.34
Weight (kg)	57 ± 9	52 ± 6	0.08
Education (years)	13 ± 2	14 ± 2	0.14
Alcohol consumption (years × g/week)	135 ± 263	83 ± 221	0.47
Left-handed (%)	1 (3)	0 (0)	

Values are expressed as means ± SD, or numbers (%).

*P* values are derived from *t*-tests for continuous variables.

Abbreviations: Pt, Patients; HC, healthy controls.

**Table 2:** Surgery and postoperative data in the patient group.

	Pt; *n* = 32
Breast cancer stage 0 or 1 (%)	22 (69)
Total mastectomy (%)	11 (34)
Duration of operation (min)	136 ± 37
Duration of anesthesia (min)	180 ± 38
Dose of propofol (mg)	106 ± 17^a^
Dose of sevoflurane (mL)	59 ± 19^b^
Dose of fentanyl (mg)	0.28 ± 0.05^b^
Post-operative hospital stay (day)	8.9 ± 1.4

Values are expressed as mean ± SD, or numbers (%).

Abbreviations: Pt, Patients.

^a^ One outlier was excluded.

^b^ Two outliers were excluded.

### Psychological assessment

A significant group (Pt/HC) by time (pre/post) interaction was observed in the DCAT1 scores, indicating poor attentional performance in the patient group after surgery (Table 3). Although the attentional scores in the Pt group showed no significant decline. However, the significant group by time interaction provides sufficient evidence of cognitive decline as a lack of learning effect [44,45].

**Table 3:** Summary of neuropsychological assessment.

		Pt; *n* = 30		HC; *n* = 19		*P* value
		Pre	Post	Pre (base line)	Post (1 week)	
Attention						
	D-CAT1	29.2 ± 5.2	31.4 ± 5.1	29.5 ± 5.4	35.5 ± 5.8	0.001 *
	D-CAT2	45.5 ± 8.1	48.0 ± 6.3	52.4 ± 9.9	48.8 ± 7.9	0.024
	D-CAT3	50.3 ± 11.0	53.3 ± 9.7	59.0 ± 14.6	61.6 ± 12.2	0.28
Processing speed						
	Stroop 1	56.4 ± 10.3	57.9 ± 9.9	58.3 ± 9.7	63.2 ± 9.1	0.01
	Stroop 3	39.9 ± 7.5	42.0 ± 7.2	46.1 ± 7.9	42.8 ± 6.3	0.005
	Digit symbol cording	68.8 ± 18.1	74.5 ± 18.2	77.5 ± 12.3	87.3 ± 14.1	0.06
Memory						
	Immediate story recall	20.9 ± 6.9	29.8 ± 7.2	24.2 ± 4.7	29.5 ± 5.1	0.033
	Delayed story recall	16.3 ± 6.7	26.3 ± 8.5	20.1 ± 5.3	27.4 ± 6.0	0.27
Executive function						
	Stroop 2	48.6 ± 9.3	50.8 ± 9.6	53.0 ± 9.1	53.8 ± 8.2	0.60
	Stroop 4	33.8 ± 8.7	34.8 ± 9.8	40.0 ± 11.4	41.2 ± 10.3	0.27
Working memory						
	Digit span (backward)	5.9 ± 2.1	6.3 ± 2.4	7.0 ± 1.6	7.7 ± 1.8	0.17
Quality of life						
	WHO-QOL 26 (average overall)	6.2 ± 0.8	6.3 ± 1.4	7.2 ± 1.2	7.2 ± 1.6	0.84

Values are expressed as means ± SD.

*P* values express the between-group significance after 1 week, and were derived from ANCOVA models.

*Significant at *P* <0.0045 using Bonferroni correction in cognitive tests.

Abbreviations: Pt, Patients; HC, healthy controls; Pre, before surgery or baseline; Post, after surgery or 1 week after baseline; D-CAT, digit cancellation task; WHO QOL, World Health Organization quality of life.

### Psychological test scores and anesthesia doses

**Fig 2:**
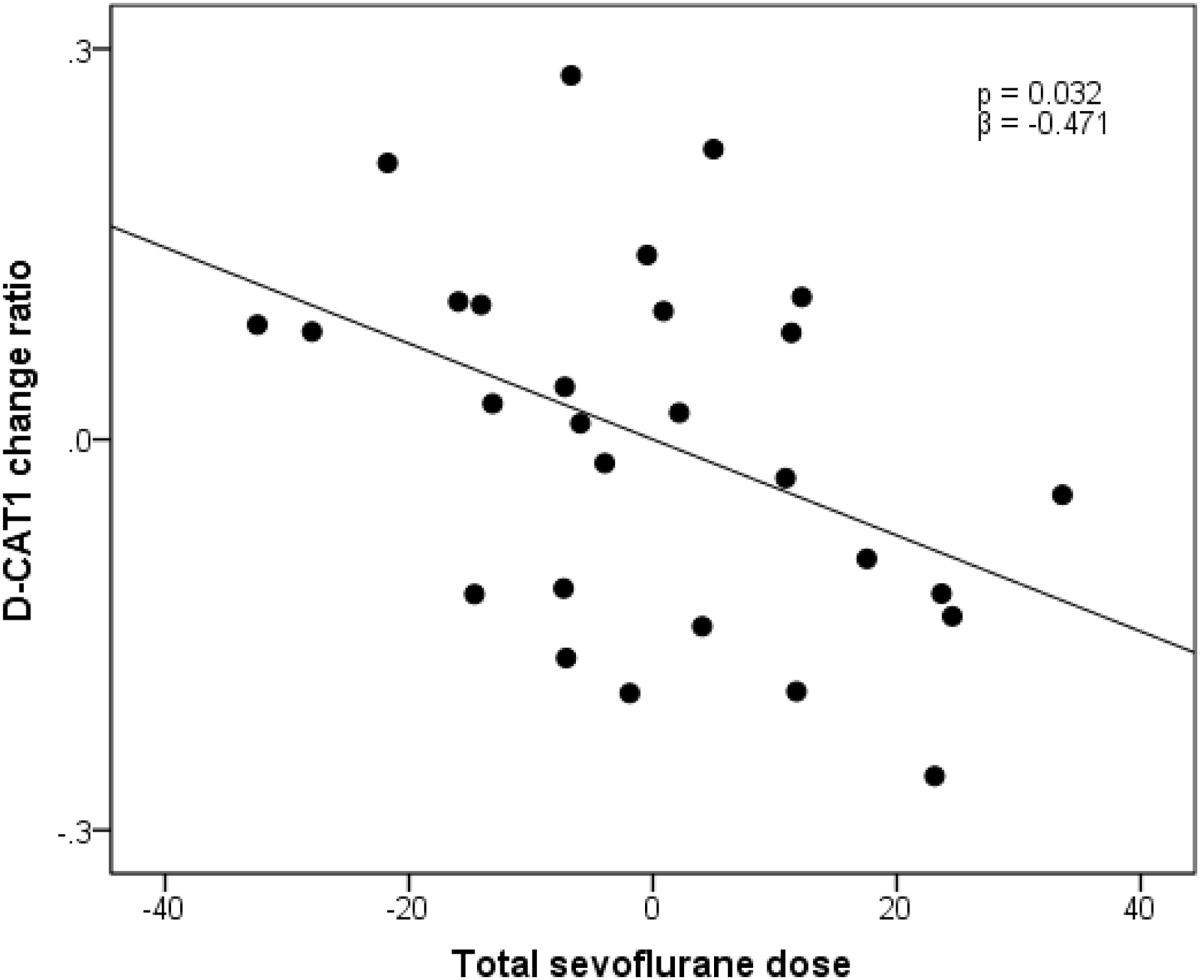
Association between attention (D-CAT1) and anesthesia (sevoflurane) in the patient group. Residual plots with trend lines depicting the correlations between residuals in the multiple regression analyses, using the change ratio of D-CAT1 scores as the dependent variable, and the total dose of sevoflurane and other confounding factors as independent variables. Two patients who did not receive post-operative examination and 2 patients who were assigned as outliers of the sevoflurane dose were removed from the analysis. Therefore the data from 28 patients were analyzed. Abbreviations: D-CAT, digit cancellation task.

After controlling for age, weight, and height, sevoflurane dose was significantly associated with the change ratio of attention score (DCAT1) in the multiple regression analysis (r^2^ = 0.247, β = −0.471, *P* = 0.032; Fig 2). In contrast, there was no significant association between the change ratio of attention score (DCAT1) and fentanyl dose (*r* ^2^ = 0.186, *P* = 0.111).

### rGMV differences

**Fig 3:**
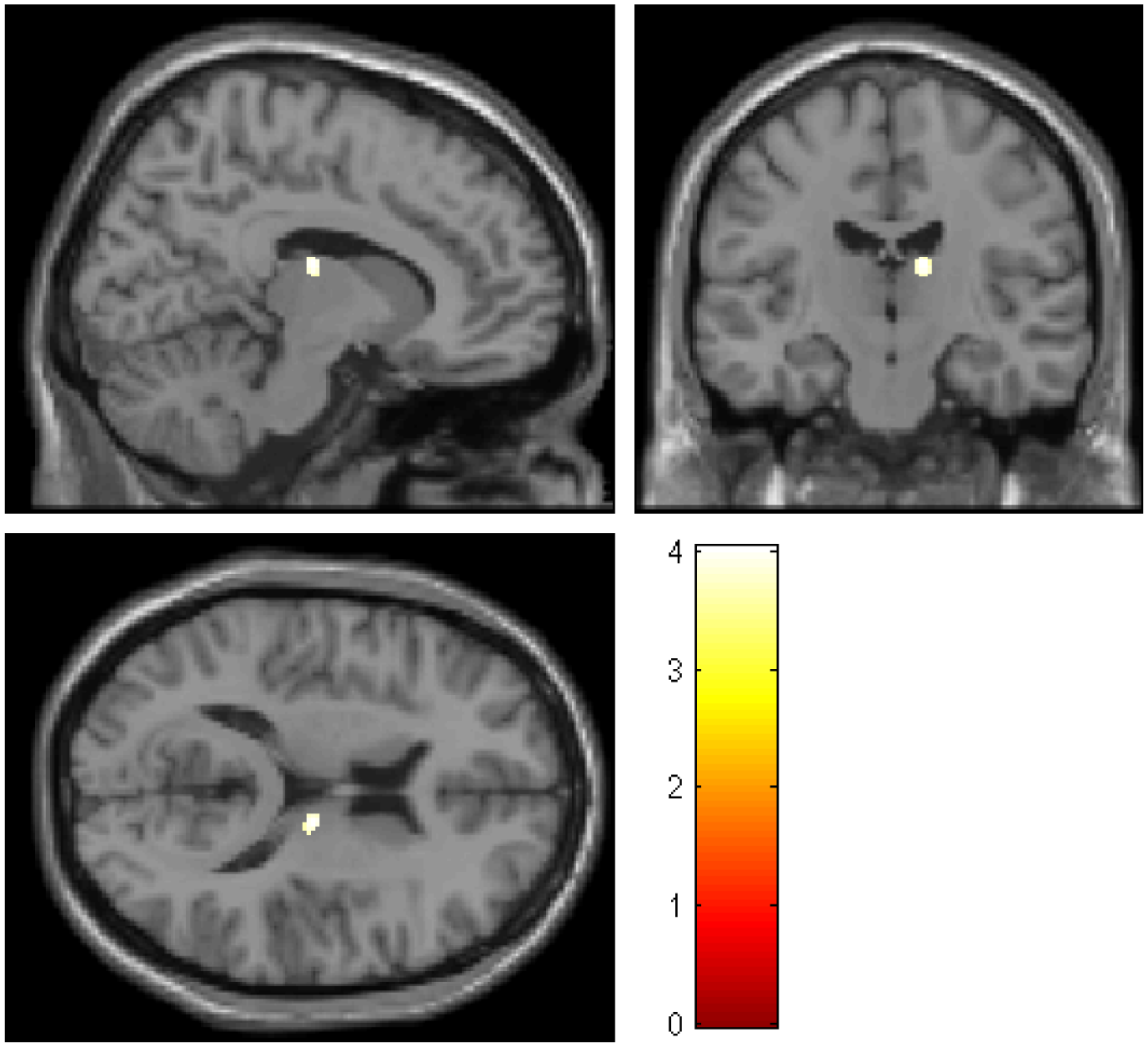
Regions showing the significant group (Pt/HC) by time (pre/post) interactions (right thalamus; *p* < 0.05, SVC). The colored bar shows the scale of the t-value. Abbreviations: Rt, right; Pt, patients; HC, healthy controls; Pre, before surgery; Post, after surgery.

Among the areas with a priori hypothesis related to anesthesia, a significant group (Pt/HC) by time (pre/post) interaction was found in the right thalamus (x = 12, y = −16, z = 15; *P* < 0.05, SVC; Fig 3). The mean pre-rGMV at the peak voxel in each cluster was 0.178 ± 0.028, and the mean post-rGMV was 0.156 ± 0.029 in the Pt group. The mean pre-rGMV at the peak voxel was 0.156 ± 0.033, and the mean post-rGMV was 0.191 ± 0.035 in the HC group.

### Psychological test scores and rGMV in the Pt

We found a marginally significant correlation between the change ratio of one attention domain subtest (D-CAT1) and post−pre thalamic rGMV (x = 8, y = −12, z = 7; *P* = 0.07, SVC).

### Anesthesia and rGMV in the Pt

There was no significant association between the anesthesia (sevoflurane) and the reduction in rGMV in the thalamus.

## Discussion

This was the first study to investigate brain structural change during the early phase responses to a low invasive surgery. Consistent with our hypothesis, we found rGMV reduction in the thalamus, which was a priori ROI given its association with anesthesia, soon after breast cancer surgery. These responses were thought to be caused by general anesthesia because other POCD risk factors, such as pre-existing cognitive impairments, systemic inflammation, and the occurrence of complications, were controlled. In addition, the Pt group showed a significant decrease in the learning effect on one subtest of the attention domain compared with the HC group. Furthermore, sevoflurane dose was significantly associated with changes in the attention score in the Pt group. Although the association between changes in thalamic rGMV and Pt attention scores did not reach the expected threshold, the correlation was marginally significant.

Our findings suggest that changes in attention and brain structures, particularly in the thalamus, may occur after surgery and may be associated with attentional dysfunction. This finding is consistent with those from previous longitudinal studies [10,46]. Attention is a fundamental cognitive function, and is associated with many other cognitive functions, including memory [47], processing speed [48], and working memory [49]. Among the subscales of the D-CAT battery, the D-CAT1 requires only sustained attention, whereas the D-CAT2 and D-CAT3 require the additional ability of selective attention and working memory [50]. Therefore, it was assumed that the purest attentional change would be detected using this subscale. In addition, the thalamus plays a fundamental role in cognitive functions, such as coordinating information flow in the brain, integrating broad cognitive processes [26,27] including incoming sensory impulses of pain [28], and regulating arousal and sleep [29,30]. As for attentional domain, the thalamus is associated with top-down modulation of attention with the frontal and parietal (usually right-lateralized) cortices [51,52]. Also, positron emission tomography (PET) study of healthy volunteers revealed activation of brain networks, including the thalamus, during an attentional orientation task [53]. With regard to specific thalamic regions, we observed a reduction in the volume of the lateral dorsal thalamus, and volume changes in the medial dorsal thalamus were correlated with changes in attention. The lateral dorsal thalamus exhibits connectivity with the precuneus [54], which plays a central part in the default mode network (DMN) [55]. Because the activity of DMN competes with those associated with attention for external stimuli [55], it is possible that the lateral dorsal part contributes to attentional dysfunction via the precuneus. On the other hand, the medial dorsal thalamus is strongly connected with the prefrontal cortex [56], which plays a critical role in attentional control [57] and may be involved in attentional dysfunction.

Our findings, together with those from previous studies, indicate that the attentional dysfunction and reduced thalamic volume observed in patients may represent an intermediate phenotype of POCD that manifest without broad cognitive dysfunction. Moreover, we believe that patients with suchl changes are at risk for POCD. Previous clinical studies have observed a high incidence of POCD soon after surgery, but rapid attenuation of these symptoms as time progressed [13], and a review of the risk factors for POCD determined that they result in multiple types of cognitive dysfunction [10]. In contrast, pre-clinical studies have demonstrated that each individual risk factor, including general anesthesia and systemic inflammation, can cause cognitive decline in particular domains under well-controlled experimental settings [16,17]. Based on these findings, it was assumed that all of the patients who had undergone surgery with general anesthesia were similarly affected by the anesthesia and that a majority of these patients would fully recover after surgery in the absence of exposure to other POCD risk factors. However, if other risk factors such as advanced age, long duration of anesthesia, high severity of the surgery, post-surgical complications, and pre-existing cognitive impairments were present, then some of the patients would develop clinical POCD with multiple and broad types of cognitive dysfunction [12–14].

A lot of knowledge about a relationship between general anesthesia and thalamus support our assumption about the short-term effect of anesthesia on POCD. A number of studies demonstrated the impact of anesthesia on the brain. Neurotoxicity of anesthesia, including sevoflurane, has been reported in animal studies to induce neuronal cell death [18,58,59]. In human studies, it was demonstrated that the thalamus is a key target for the actions of anesthetics [60]. In addition, anesthetics (including sevoflurane) reduced regional cerebral blood flow [22] and regional glucose metabolism [25] in the thalamus. Additionally, the potential involvement of anesthesia in the development of POCD is a much-debated topic. Some studies have indicated that the risk of POCD increases when general anesthesia is used [61], but a meta-analysis examining the relationship between type of anesthesia (regional or general) and POCD concluded that general anesthesia does not contribute to long-term POCD [62]. Our findings indicate that general anesthesia is a risk factor for POCD in the early postoperative period. However, it is likely that additional risk factors are necessary for the development of POCD. Thus, compared with other risk factors, the effect of general anesthesia may be too subtle to contribute to long-term POCD.

Our study had several strengths and limitations that should be discussed. The strengths of this study included controlling a number of risk factors for POCD, the prospective cohort design, a uniform protocol of MRI scanning at two time points, and the homogeneity of lower surgical stress. According to these strengths, we successfully detected thalamic volume reduction and attentional dysfunction as an intermediate phenotype of POCD. Limitations of this study included the relatively small sample size, which might have prevented the detection of more subtle effects on cognitive function. Another limitation of the present study is that the significant group-by-time interaction in the right thalamus was based not only on decreased thalamic volume in the patient group but also on increased thalamic volume in the control group. We conclude that the short-term intervention with psychological tests was responsible for the increased rGMV in the HCs. A previous study found significant increases in rGMV following a very short-term intervention (90 min at most) [63]. Thus, it is possible that a short-term intervention of several psychological tests (about 2 h) relevant to the learning effects observed in the psychological measures could have caused structural changes in the brains of HCs. Thus, we believe that our finding of altered thalamic volume after surgery was reliable. Moreover, we could not completely exclude the effects of inflammation. We did not measure inflammatory factors to estimate effects of inflammation. However, a previous animal study found that low invasive surgery did not impair hippocampal-dependent cognitive function [64], which is thought to be highly vulnerable to inflammation [65]. Thus, inflammation-mediated changes were unlikely to have contributed to cognitive dysfunction in patients after low invasive surgery, such as that for breast cancer. Finally, because there was no direct relationship between the amount of anesthetic used and the change in thalamic volume, it was not possible to conclude whether the anesthesia could have contributed to this reduction based on the present findings. However, a significant amount of previous evidence supports this relationship [22–25].

Despite these limitations, we detected significant changes in cognitive function and brain structure in the early postoperative stage. In general, the clinical symptoms of POCD are too heterogeneous to accurately clarify its neuropathology, but the present findings can provide new insights for future studies aiming to improve the QOL of patients who receive surgery.

## Supporting Information

S1 Text Study protocol. (DOCX)

S2 Text IRB protocol. (DOCX)

S3 Text Certification of approval. (PDF)

S1 Table TREND statement checklist. (PDF)
