# Supplementary material for: Postoperative Structural Brain Changes and Cognitive Dysfunction in Patients with Breast Cancer
Source: PLoS One. 2015 Nov 4;10(11):e0140655. doi: 10.1371/journal.pone.0140655 (PMC4633203; doi:10.1371/journal.pone.0140655)
Supplement: S1 Text — (DOCX) [file pone.0140655.s001.docx]

**Neurological underpinnings of cognitive dysfunctions in breast cancer patients**

**: a longitudinal observational study.**

**Trial Protocol**

| **Title** | Neurological underpinnings of cognitive dysfunctions in breast cancer patients |
| --- | --- |
| **Condition** | **Condition:** breast cancer  **Classification by specialty**: Breast surgery/ Psychiatry/ Anesthesiology **Classification by malignancy**: Malignancy  **Genomic information**: NO |
| **Objectives** | **Narrative objectives1**: The aim of this study is to clarify the influence of operations and hormonal therapy on breast cancer patients' cognitive functions and brain structure.  **Basic objectives2**: Others  **Basic objectives –Others** : To clarify the influence of breast cancer therapy.  **Trial characteristics_1**: Confirmatory  **Trial characteristics_2**: Pragmatic  **Developmental phase**: Not applicable |
| **Assessment** | **Primary outcomes:** Cognitive function and brain structures measured by MRI  Cognitive measures:  1) Digit Cancellation Task (D-CAT)  2) digit symbol coding  3) Logical Memory, immediate and delay  4) Stroop test  5) digital span – backward  **Key secondary outcomes:** Psychological measurement |
| **Base** | **Study type:** Observational |
| **Eligibility** | **Age-lower limit**: Not applicable  **Age-upper limit**: 80 years-old />=  **Gender**: Female  **Key inclusion criteria**: Breast cancer patient, postmenopausal  and no history of neoadjuvant therapy  **Key exclusion criteria**: Previous history of cancer therapy with drug, metastatic cancer, history of any neurological condition, traumatic brain injury, mental retardation, use of psychotropic medication, drug or alcohol abuse, or any contraindication to undergoing on MRI use  **Target sample size**: Since this is the first clinical trial of its kind in a brain structural research field, no a priori sample size estimation was carried out. |
| **Setting** | **Setting** : Sendai city, Miyagi prefecture in Japan |
| **Research contact person** | **Name of lead principal investigator**: Ryuta Kawashima  **Organization**: Institute of development, aging and cancer, Tohoku university  **Division name**: Dept. Functional brain imaging  **Address** : Seiryo-cho 4-1 Aoba-ku Sendai-shi Miyagi-ken Japan  **TEL**: 022-717-7988  **Email**: [ryuta@idac.tohoku.ac.jp](mailto:ryuta@idac.tohoku.ac.jp) |
| **Public contact** | **Name of contact person**: Chiho Sato  **Organization**: Institute of development, aging and cancer, Tohoku university  **Division name**: dept. Functional brain imaging  **Address** : Seiryo-cho 4-1 Aoba-ku Sendai-shi Miyagi-ken Japan  **TEL**: 022-717-7988  **Email**: [schiho@idac.tohoku.ac.jp](mailto:schiho@idac.tohoku.ac.jp) |
| **Sponsor** | **Name of primary sponsor**: Institute of development, aging and cancer, Tohoku University |
| **Funding Source** | **Source of funding**: Institute of development, aging and cancer, Tohoku University  A.S. is supported by a Grant-in-Aid for Young Scientists (B) (KAKENHI 24790653) from the Ministry of Education, Culture, Sports, Science and Technology.  **Category of Org** : Self funding  **Nation of funding**: Japan |
| **Collaborators** | Ryuta Kawashima ^(1,4,6)^, Atsushi Sekiguchi ^(1,3)^, Masaaki Kawai ^(2)^, Yuka Kotozaki ^(4)^, Rui Nouchi ^(4,5)^, Hiroshi Tada ^(2)^, Hikaru Takeuchi ^(6)^, Takanori Ishida ^(2)^, Yasuyuki Taki ^(3,6,7)^, and Noriaki Ohuchi ^(2)^  (1) Department of Functional Brain Imaging, Institute of Development, Aging and Cancer (IDAC), Tohoku University, Sendai, Japan  (2) Department of Surgical Oncology, Tohoku University, Sendai, Japan  (3) Division of Medical Neuroimage Analysis, Department of Community Medical Supports, Tohoku Medical Megabank Organization, Tohoku University, Sendai, Japan  (4) Department of Advanced Brain Science, Smart Ageing International Research Center, IDAC, Tohoku University, Sendai, Japan  (5) Human and Social Response Research Division, International Research Institute of Disaster Science, Tohoku University  (6) Division of Developmental Cognitive Neuroscience, IDAC, Tohoku University, Sendai, Japan  (7) Department of Nuclear Medicine and Radiology, Institute of Development, Aging and Cancer, Tohoku University, Sendai, Japan |
