# Supplementary material for: Postoperative Structural Brain Changes and Cognitive Dysfunction in Patients with Breast Cancer
Source: PLoS One. 2015 Nov 4;10(11):e0140655. doi: 10.1371/journal.pone.0140655 (PMC4633203; doi:10.1371/journal.pone.0140655)
Supplement: S2 Text — (DOCX) [file pone.0140655.s002.docx]

An application for the ethics committee

**Title of the study**

Neurological underpinnings of cognitive dysfunctions in breast cancer patients

**The principal investigator**

Name Ryuta Kawashima

Position Prof.

Name of department Functional brain imaging

Name of institute Institute of development, aging and cancer, Tohoku university

The ethics committee course already attended

**Type of the study**

Intervention study except pharmaceutical products and medical device

**Type of the study form**

Multi-international joint research No

**Study period**

April 2011 ~ March 2016

**Collaborators**

Noriaki Ohuchi Department of Surgical Oncology, Tohoku University,

Takanori Ishida Department of Surgical Oncology, Tohoku University,

Hiroshi Tada Department of Surgical Oncology, Tohoku University,

Masaaki Kawai Department of Surgical Oncology, Tohoku University,

Yasuyuki Taki Division of Medical Neuroimage Analysis, Department of Community Medical Supports, Tohoku Medical Megabank Organization (TOMMO), Tohoku University

Kanno Akitake Department of Functional Brain Imaging, Institute of Development, Aging and Cancer (IDAC), Tohoku University

Hikaru Takeuchi Division of Developmental Cognitive Neuroscience, IDAC, Tohoku University

Atsushi Sekiguchi Division of Medical Neuroimage Analysis, Department of Community Medical Supports, TOMMO, Tohoku University

Rui Nouchi Department of Advanced Brain Science, Smart Ageing International Research Center, IDAC, Tohoku University

Yuka Kotozaki Department of Advanced Brain Science, Smart Ageing International Research Center, IDAC, Tohoku University

Atsushi Sakuma Department of Functional Brain Imaging, IDAC, Tohoku University

Chiho Sato Department of Surgical Oncology, Tohoku University

1. **Title of the study**

Neurological underpinnings of cognitive dysfunctions in breast cancer patients

1. **Study outline**

This protocol is revised version approved by the Ethics Committee of the Tohoku University Graduate School of Medicine. We have already started this study from April 2011. Before we started the intervention part of this study, we add the protocol more concrete plan of the intervention.

- 1. **Background**


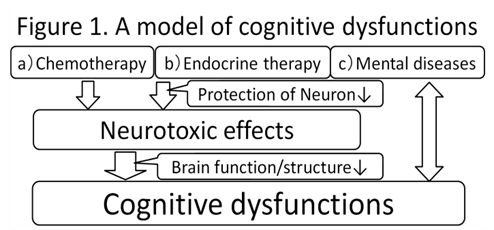

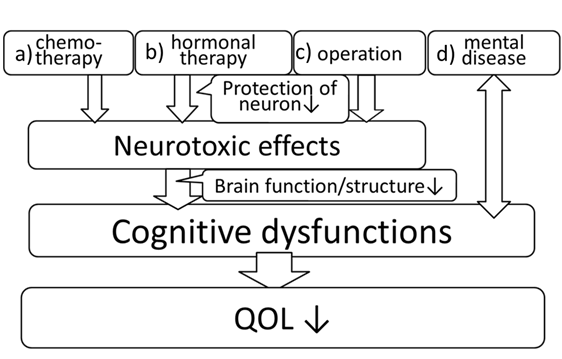
Recently, cognitive dysfunctions (impairments of memory, attention, and execution functions) in patients with breast cancer have been given attention due to improvements in their prognosis (Taillibert 2007). Cognitive dysfunctions can impair patient’s QOL and decrease the dose-intensity of anti-cancer drugs (Taillibert 2007), which can worsen a patient's prognosis (Budham 1998, Lis 2007). Past studies report that cognitive dysfunctions in patients with breast cancer are thought to originate in neuron injuries caused by anti-cancer drugs, which are represented by abnormalities of specific brain structures and brain functions (McDonald 2010, Ruiter 2010). On the other hand, endocrine therapy, which comprises controlling the functions of estrogen, has become a major treatment of breast cancer. Since estrogen protects neuronal cells (Bruce 2002), endocrine therapy may also injure neurons indirectly. Although recent studies revealed that endocrine therapy causes cognitive dysfunctions (Phillips 2010), there is no neuroimaging study for investigating the neural background of cognitive dysfunction caused by endocrine therapy. Moreover, the experience of surgical operation also causes cognitive dysfunction (Newfield 2009) and the postoperative cognitive dysfunction related to mortality (Steinmetz 2009).　Anesthesia reported to have neuro toxicity (Hudson 2011). In addition, mental diseases such as adjustment disorder, major depression, delirium, and posttraumatic stress disorder (PTSD) are likely to coincide with the breast cancer (Akechi 2001, Matsuoka 2002). It is widely known that these mental diseases also cause cognitive dysfunction. One unresolved issue is how these three factors [i.e. a) chemotherapy, b) endocrine therapy, c) operation, and d) mental diseases (Fig. 1)] affect cognitive dysfunctions in patients with breast cancer. We would like to focus on the neural underpinnings of these three factors causing cognitive dysfunctions.

The purpose of the present study is to clarify the influence of the three factors that can cause cognitive dysfunctions in patients with breast cancer, and to open the door to finding appropriate preventions and treatments for breast cancer. We hypothesized that we would be able to create a new model of cognitive dysfunctions in patients with breast cancer which would include the three factors mentioned above (Fig.1).

- 1. **Objective**

The objective of the research is to clarify the influence of the three factors (chemotherapy, endocrine therapy, and mental diseases) that cause cognitive dysfunctions in patients with breast cancer, and to open the door to finding appropriate preventions and treatments for breast cancer.

- 1. **Ethics**

The MRI machine uses magnetic waves to take images of the body’s interior and does not emit radioactive waves. However, if participants fall under any of the below categories, we will not allow to undergo the tests in line with safety:

If participants have any metal contained within your body (e.g. brain aneurysm clips, cardiac pacemakers, orthopaedic hardware such as bolts or plates, are undergoing dental work, have dyed your hair within the past month, have tattoos, etc.), have extreme myopia (are extremely short-sighted), have a hearing disability, are claustrophobic, left-handed, could possibly be pregnant, are a frequent drug user, or are wearing winter outfits with new materials (e.g. Thermal clothing), we will exclude them for their safety.

In addition, we will provide the accident insurance, in case accidents occur during MRI examinations. We will explain well that participants can stop the examination immediately in case they experience bad health conditions.

MEG and EEG are non-invasive examination, and there are no reports about health damage with MEG and EEG. We will explain well that participants can stop the examination immediately in case they experience bad health conditions.

All personal data will be kept in strict confidence by using linkable anonymous.

We think the study is well ethical with the countermeasures we explained above.

- 1. **Participant**

Patients with breast cancer from Tohoku University Hospital: 150 subjects

Age-matched healthy controls: 60 subjects

- 1. **Study protocol**


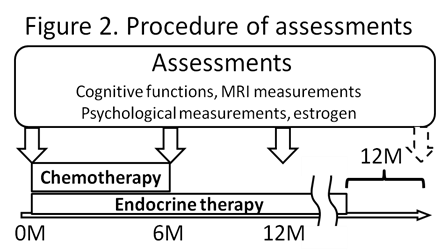
We will assess the measurements at three time points at least: just before and after surgical operation (0th month), after the 6th month (just after end of chemotherapy), and after the 12th month. (Fig. 2)

In the present study, we plan to recruit patients with breast cancer from Tohoku University Hospital. We will divide patients into 4 groups; the Endocrine therapy group, the Chemotherapy group, the Endocrine and chemotherapy group, and non-therapy group. We expect to recruit 30 subjects for each group. Adding an age-matched healthy control group, we will measure 1) cognitive function (attention, verbal frequency, declarative memory, working memory, executive function, fluid intelligence and so on), 2) brain structures (MPRAGE for gray matter/white matter volume, diffusion tensor imaging for white matter integrity, and myelination transfer ratio) and brain functions (emotional processing, the working memory task, and so on) using MRI, 3) psychological measurements (depression, anxiety, self-esteem, gender role, quality of life and so on), and 4) serum estrogen levels. In addition, we will perform a medical interview (the Mini-International Neuropsychiatric Interview; MINI) for each subject to diagnose mental diseases.

We will calculate the change rate of each parameter (i.e. cognitive functions, MRI data, psychological measurements, and serum estrogen level) at the three time points. Next, we will perform multiple regression analyses using the change rates of the cognitive dysfunctions as dependent variables and other parameters as independent variables to detect the explanatory factors of the cognitive dysfunctions. Moreover, we will conduct a path analysis to verify our model of cognitive dysfunctions in patients with breast cancer.

As a next step, we plan to develop a new intervention program to improve the cognitive dysfunctions from a neurological standpoint. We chose working memory (WM) training for our intervention training program (Jaeggi 2008, Takeuchi 2010). Our WM training is dual task training. A dual task training requires an individual to perform two tasks simultaneously. For example, in the n-back task, the subject is presented with a sequence of stimuli, and the task consists of indicating when the current stimulus matches the one from n steps earlier in the sequence. The load factor n can be adjusted to make the task more or less difficult. We chose “ONI training” which is played with Nintendo 3 DS for our intervention program. “ONI training consists of 8 kinds of working memory programs (for example, the n-back test, reading or listening span tests etc.). It is advantageous because the combination of components involved in training tasks is important in the observed transfer effect (Green 2008). Participants can continue at an optimal task level using the computer program. This intervention study is performed by RCT. We chose “Tetris” for control task. The validity of “Tetris” as a control task is demonstrated (Nouchi 2012). The duration of this intervention is 8 week. We will evaluate the intervention program as same way to above explained.

- 1. **Funding source**

Operating support funds

A Grant-in-Aid for Young Scientists (B) (KAKENHI 24790653) from the Ministry of Education, Culture, Sports, Science and Technology

1. **Setting**

Institute of development, aging and cancer, Tohoku University

1. **Ethical considerations**
   1. **Protection of personal information**

The data collected via these tests is for research purposes only. We will ensure the privacy of all information and data regarding these tests, and will be kept in strict confidence.

All data will be marked with individual IDs and will not contain any personal information at all. Data analysis is handled anonymously using these IDs.

Researchers are able to obtain the de-identified brain imaging data and demographic data including age, weight, and height, when they make a request to verify the results of the current research. All personal information is strictly protected. We don’t put any data in public domains, to prevent the possibility of a third party identifying the individual. We obtain informed consent from each participant before making the de-identified data available in a public domain.

- 1. **Selection criteria**

Rewards Yes

- 1. **Use of research data**

All personal data will be kept in strict confidence under the direction of the data controller (Takanori Ishida, MD Department of Surgical Oncology, Tohoku University).

All personal information will be kept anonymous. (Linkable anonymizing)

The cross-reference of the IDs and the personal information will be kept at a lockable storage unit in the research center and we will not release the personal information to anyone other than the researchers associated to this research. When this study finishes, the cross-reference of the IDs and the personal information will be discarded.

- 1. **Informed consent**

We will provide an informed consent by written and spoken, and obtain a signed agreement form.

- 1. **In the case that the participants don’t have enough judgment**

Not applicable

1. **Expected results**

The expected outcome of the research is that different factors will cause different cognitive dysfunctions represented by abnormalities of different brain structures and brain functions. We hope we will be able to detect cognitive dysfunctions represented by brain regions such as the dorsolateral prefrontal cortices and characterized by high neural plasticity so that we will be able to design a new cognitive rehabilitation program to improve these dysfunctions.

1. **Expected disadvantage**
   1. **Measures taken to preserve safety**

As for the dangerousness wearing and containing metals with the body in the MRI examination, we confirm that the participants don’t wear or contain metals with their body (e.g. brain aneurysm clips, cardiac pacemakers, orthopaedic hardware such as bolts or plates, are undergoing dental work, have dyed your hair within the past month, have tattoos, etc.) by written and spoken several times.

As for the dangerousness of flying metals in the MRI examinations because of its powerful magnetic field, we never carry metals in the MRI room, and the MRI examinations are conducted by enough experienced person for the safety.

- 1. **Measures taken against accident**

Study type: Intervention study except pharmaceutical products and medical device

Accident insurance: Yes

1. **Data after the completion of research period**

Preserve: Yes

The brain imaging data and neuropsychological data will be kept at a lockable storage unit in the research center.

Data controller: Takanori Ishida, MD, Department of Surgical Oncology, Tohoku University

Anonymous: unlinkable anonymize

1. Notes

Conflict of interest: Our study is conducted using a grant (A Grant-in-Aid for Scientists (KAKENHI) from the Ministry of Education, Culture, Sports, Science and Technology etc.).

Ryuta Kawashima, the principal investigator, receives the royalty income which is over 2 million yen from Nintendo Inc. “Nintendo 3DS”, “ONI training”, “Tetris” which are used in this study are products of Nintendo Inc.

Our study is screened and approved by Conflict of Interest Committee (COIC). If its interest changes, we will be checked by the COIC every time. We keep fairness of our study between corporations.

We established an evaluation committee for our study.

Contact address: Business – Academia Collaboration Study Evaluation Committee, IDAC, Tohoku University

980 – 8575

Seiryo-cho 4 – 1 Aoba-ku Sendai-shi, Miyagi, Japan

TEL 022 – 717 – 8445 FAX 022 – 717 – 8452
