# Supplementary material for: Postoperative Structural Brain Changes and Cognitive Dysfunction in Patients with Breast Cancer
Source: PLoS One. 2015 Nov 4;10(11):e0140655. doi: 10.1371/journal.pone.0140655 (PMC4633203; doi:10.1371/journal.pone.0140655)
Supplement: S3 Text — (PDF) [file pone.0140655.s003.pdf]

Certificate of Approval

Ethics Committee

Tohoku University Graduate School of Medicine

The following protocol was approved:

Title of Protocol
Neurological underpinnings of cognitive dysfunctions in breast cancer patients

Protocol Identification Number
2014-1-826

Principal Investigator
Ryuta Kawashima

Period of Research
April, 2011 ~ March, 2016

Comments

Name : Tooru Shimosegawa
Title : Dean, Tohoku University Graduate School of Medicine

Tooru Shimosegawa　　April 20, 2015
Signature　　Date

Name : Atsushi Asai
Title : Chair
Ethics Committee of Tohoku University Graduate School of Medicine

Atsushi Asai　　April 20, 2015
Signature　　Date
